# Supplementary material for: The Systems Biology Research Tool: evolvable open-source software
Source: BMC Syst Biol. 2008 Jun 29;2:55. doi: 10.1186/1752-0509-2-55 (PMC2446383; doi:10.1186/1752-0509-2-55)
Supplement: Additional file 1 — SBRT Archive. An archive of the current version of the Systems Biology Research Tool. [file 1752-0509-2-55-S1.zip › sbrt-1.4.0/doc/developers_guide/api/sbrt/kernel/io/class-use/WarningHandler.html]

Uses of Interface sbrt.kernel.io.WarningHandler


|  |  |  |
| --- | --- | --- |
| **Overview** \| **Package** \| **Class** \| **Use** \| **Tree** \| **Deprecated** \| **Index** \| **Help** | |  |
| PREV   NEXT | **FRAMES**    **NO FRAMES**     **All Classes** |  |


---


## **Uses of Interface sbrt.kernel.io.WarningHandler**

| Packages that use WarningHandler | |
| --- | --- |
| **sbrt.kernel.io** | Provides classes and interfaces used to perform kernel-level I/O. |

Uses of WarningHandler in sbrt.kernel.io

| Methods in sbrt.kernel.io that return WarningHandler | |
| --- | --- |
| `static WarningHandler` | `WarningHandlerFactory.getDefault()`             Returns the default warning handler. |

---


|  |  |  |
| --- | --- | --- |
| **Overview** \| **Package** \| **Class** \| **Use** \| **Tree** \| **Deprecated** \| **Index** \| **Help** | |  |
| PREV   NEXT | **FRAMES**    **NO FRAMES**     **All Classes** |  |


---
